# A Single Dose of Prednisolone as a Modulator of Undercarboxylated Osteocalcin and Insulin Sensitivity Post-Exercise in Healthy Young Men: A Study Protocol

**DOI:** 10.2196/resprot.5119

**Published:** 2016-06-03

**Authors:** Itamar Levinger, Tara C Brennan-Speranza, Nigel K Stepto, George Jerums, Lewan Parker, Glenn K McConell, Mitchell Anderson, Andrew Garnham, David L Hare, Peter R Ebeling, Ego Seeman

**Affiliations:** ^1^Clinical Exercise Science Research ProgramInstitute of Sport, Exercise and Active Living (ISEAL)Victoria UniversityMelbourneAustralia; ^2^Department of PhysiologyBosch Institute for Medical ResearchUniversity of SydneySydneyAustralia; ^3^University of Melbourne and the Department of EndocrinologyAustin HealthMelbourneAustralia; ^4^School of Exercise & Nutrition SciencesDeakin UniversityMelbourneAustralia; ^5^University of Melbourne and the Department of CardiologyAustin HealthMelbourneAustralia; ^6^Department of Medicine, School of Clinical Sciences,Faculty of Medicine, Nursing and Health SciencesMonash UniversityMelbourneAustralia

**Keywords:** bone metabolism, exercise, glycaemic control, undercarboxylated osteocalcin, prednisolone

## Abstract

**Background:**

Undercarboxylated osteocalcin (ucOC) increases insulin sensitivity in mice. In humans, data are supportive, but the studies are mostly cross-sectional. Exercise increases whole-body insulin sensitivity, in part via ucOC, while acute glucocorticoid treatment suppresses ucOC in humans and mice.

**Objectives:**

A single dose of prednisolone reduces the rise in ucOC produced by exercise, which partly accounts for the failed increase in insulin sensitivity following exercise.

**Methods:**

Healthy young men (n=12) aged 18 to 40 years will be recruited. Initial assessments will include analysis of fasting blood, body composition, aerobic power (VO_2peak_), and peak heart rate. Participants will then be randomly allocated, double-blind, to a single dose of 20 mg of prednisolone or placebo. The two experimental trials will involve 30 minutes of interval exercise (90%-95% peak heart rate), followed by 3 hours of recovery and 2 hours of euglycaemic- hyperinsulinaemic clamp (insulin clamp). Seven muscle biopsies and blood samples will be obtained at rest, following exercise and post-insulin clamps.

**Results:**

The study is funded by the National Heart Foundation of Australia and Victoria University. Enrollment has already commenced and data collection will be completed in 2016.

**Conclusion:**

If the hypothesis is confirmed, the study will provide novel insights into the potential role of ucOC in insulin sensitivity in human subjects and will elucidate pathways involved in exercise-induced insulin sensitivity.

## Introduction

Insulin resistance is characterized by impaired insulin action in target tissues. Skeletal muscle is a major site of glucose uptake and disposal in response to insulin and skeletal muscle can become insulin resistant in obese individuals and those with type 2 diabetes mellitus (T2DM). In mice, the skeleton is partly involved in determining insulin secretion, insulin sensitivity, and glucose tolerance via the undercarboxylated form of osteocalcin (ucOC) [1,2]. UcOC- deficient mice have insulin resistance and in obese mice recombinant ucOC treatment reduces glucose levels and increases insulin secretion and sensitivity [2,3]. Moreover, recombinant ucOC increases muscle glucose uptake post ex vivo contraction in mice [4].

In humans, ucOC may influence glycemic control but most evidence is based on cross-sectional studies [5,6]. For example, patients with T2DM have lower serum ucOC than controls. A lower serum ucOC is associated with a higher fasting glucose and fat mass, and lower insulin sensitivity [7,8]. This suggests ucOC participates in glucose homeostasis in humans; however, prospective interventional studies are required [9].

Exercise increases insulin sensitivity in nonobese and obese subjects and in patients with T2DM independent of weight loss [10] Even a single bout of exercise increases glucose handling and insulin sensitivity [11]. Acute exercise also increases ucOC, which is associated with improved glycemic control and increased insulin sensitivity post exercise [7,12,13]. Thus, ucOC may contribute to the regulation of insulin sensitivity in humans.

To explore this, we have designed a prospective study aimed at inhibiting the rise in ucOC using a single dose of a glucocorticoid (GC). GC treatment is used regularly as an anti-inflammatory and immunosuppressive agent [14,15]. Long-term GC treatment causes bone loss, obesity, insulin resistance, and T2DM[16]. The detrimental long-term effects of GC on insulin resistance are partly mediated via osteoblasts, the bone cells responsible for the production of ucOC [14,15,17]. The reduction in insulin sensitivity following short-term GC treatment is related to an acute reduction in osteoblastic function and ucOC levels and not to the GC effects on skeletal muscle or liver, at least in mice [17]. As such, we will use a single dose of a GC, prednisolone, as a tool to acutely suppress ucOC and examine the consequent effect on whole-body insulin sensitivity and muscle metabolism, including measuring GC signaling in muscle. The regulation of GC-target genes, glucocorticoid-induced leucine zipper *(Gilz)*[18] and FK506-binding protein 5 *(Fkbp5)*[19] will be analyzed by polymerase chain reaction (PCR) and Western blot from muscle biopsies of humans with and without an acute dose of prednisolone. We hypothesize that the protein levels of these GC-targets will not be altered in the short time frame, even if gene expression is altered. Other studies in mice and humans confirm a 40% to 50% reduction in circulating OC within 12 to 24 hours after the commencement of GC treatment [20]. On the other hand, 5 days of prednisone treatment had minimal effect on muscle protein synthesis, breakdown, mitochondrial function, strength, and resting energy expenditure in men [21]. Thus, evidence from mice and humans indicates that a reduction in serum OC and ucOC following GC treatment occurs prior to muscle atrophy signaling changes in skeletal muscle. We hypothesize that insulin sensitivity will be partly reduced due to changes in insulin signaling proteins subsequent to a reduction in circulating ucOC levels.

No previous research has used GC (prednisolone) as a tool to examine the effect of ucOC on insulin sensitivity post exercise. Thus, the aim of this study is to test the hypothesis that attenuation of the increase in ucOC following exercise by a single dose of prednisolone reduces insulin sensitivity post exercise, as measured by euglycaemic-hyperinsulinaemic clamp, as well as impairing skeletal muscle insulin signaling. We also hypothesize that markers of muscle damage will not be increased by a single dose of prednisolone.

## Methods

### Study Design and Participants

This is designed as a double-blind, randomized controlled, cross-over study. We aim to recruit 12 healthy young men. Volunteers will be recruited via several advertisement strategies including flyers, global emails to staff and students from Victoria University, and Web-based advertisements.

### Criteria

Healthy young men aged 18 to 40 years, body mass index range between 19 and 27 kg/m^2^with fasting blood glucose ≤ 5.6 mmol/L^1^will be recruited for the study. Men with bone disease (such as osteoporosis), metabolic or cardiovascular disease, and/or those who are taking any medication known to affect bone metabolism, insulin secretion, or insulin sensitivity will be excluded. Also, those with a musculoskeletal and/or orthopedic condition (such as severe osteoarthritis) that prevents normal daily function (such as walking) will be excluded. Conversion of ucOC to OC is dependent on vitamin K [22]. As such, volunteers on warfarin therapy or vitamin K supplementation or restriction will be excluded.

### Sample Size and Data Analysis

The sample size is based on our previous work where exercise significantly increased ucOC by approximately 6.5% and insulin sensitivity by approximately 35% during a euglycaemic-hyperinsulinaemic clamp (n=11) [7,12]. As such, we estimate that the sample size needed in this cross-over design (power of 80%, alpha=0.05) is 12 individuals.

Descriptive statistics will be used to describe the volunteers’ characteristics as well as the study measurements. Changes from pre-to-post exercise within each trial and between trials will be analyzed by paired *t*-tests. General linear model analysis of the variance will be use to compare multiple time-points within and between interventions. Multilinear regression model will be used to determine associations between selected measurements. All data will be reported as mean ± standard error of mean and all statistical analyses will be conducted at the 95% level of significance ( *P*≤.05).

### Screening

Fasting blood test: a blood sample will be collected following an overnight fast. Blood will be analyzed at Austin Health pathology using the standard hospital assay protocols for glucose, HbA1c, insulin, triglyceride, low-density lipoprotein, and high-density lipoprotein.

Body composition: dual-energy x-ray absorptiometry (DXA) will be used to assess total body fat and lean body mass. In addition, the DXA will be used to assess fat mass in the abdominal region as well as bone mineral density to exclude osteoporosis [23,24].

Aerobic power (VO_2peak_) will be assessed during a sign and symptom-limited graded exercise test as we previously described [23]. The exercise will be performed on a cycle ergometer and VO_2_for each 15-second interval will be measured by a gas analyzer that will be calibrated as per the manufacturer’s instructions before each test. Participants will be asked to refrain from physical activity (48 hours), and alcohol and caffeine ingestion (24 hours) prior to the screening session and trial days.

### Randomization and Blinding

At the conclusion of the baseline/screening assessments volunteers will be randomly allocated (block allocation) in a double-blind fashion to determine the order of the treatments (prednisolone or placebo). Randomization will be performed using sealed opaque envelopes. The person responsible for the randomization, the investigators and the participants will not be aware of the intervention (prednisolone or placebo). The code for the randomization will be held by the medical practitioner who will supervise the insulin clamp to allow medical intervention in case of adverse responses during the exercise or insulin clamps.

### Intervention

**Figure 1:**
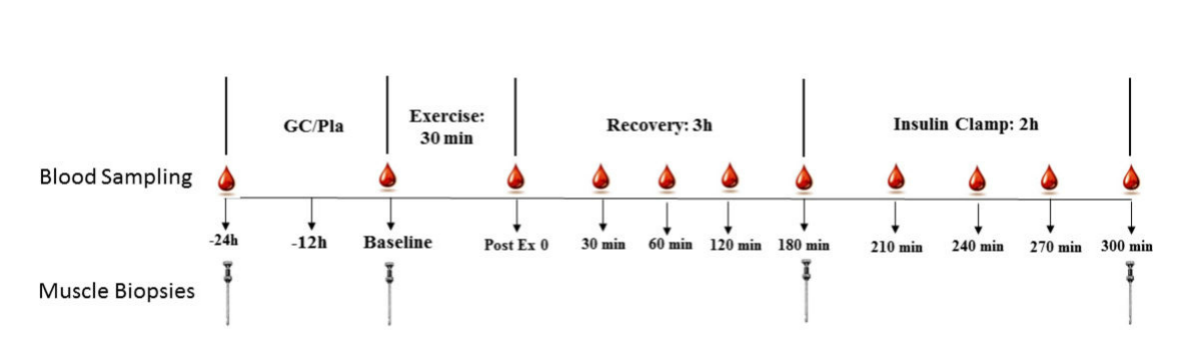
Experimental design. Participants will perform the protocol twice. Prednisolone (GC), or placebo (Pla) orally in a double-blind, randomized, cross-over design. The two experimental days will be separated by 1 to 3 weeks. The -24 hour biopsy will be obtained only once, prior to the first treatment. We will use the data from this biopsy for comparison across the 2 trial days.

Volunteers will attend our laboratory twice for the experimental trials (Figure 1). On the day prior to the first trial, participants will be asked to record their daily diet in a food diary, which will then be replicated the day prior to their second trial. The two trials (prednisolone or placebo) will be performed 1 to 3 weeks apart. This ensures that the effect of the single dose of prednisolone is “washed out” [25]. Twenty-four hours prior to the first trial day, and a minimum of 1 week after the VO_2peak_test, a single muscle biopsy and 15 ml of blood will be obtained. To avoid an additional biopsy, data from this resting biopsy will be used as the “24 hour prior” time-point for both the placebo and prednisolone trials. Then, at 7 PM (12 hours before the trial) on the day prior to each experimental trial participants will ingest a 20-mg capsule of prednisolone or placebo orally (Avicel- microcrystalline Cellulose NF PH105) (Figure 1).

The timing of the prednisolone ingestion is based on previously published data on the suppressive effect of acute GC treatment on osteocalcin [20]. The prednisolone dose that will be used in this study is slightly higher compared with the dose used by Nielsen et al [20] (10 mg), but is lower compared with doses used in clinical practice to treat inflammatory conditions (30-70 mg/day). Both the prednisolone and placebo will be purchased from the same pharmacy and look and taste identical. Avicel is widely used as a placebo in a variety of drug trials [26].

The following day (the trial day) participants will attend our laboratory in the morning (7 AM) after an overnight fast for the experimental trial (Figure 1). The participant will lie down on a bed and a cannula will be inserted to the antecubital vein. Then a resting blood sample will be obtained. In addition, a resting muscle biopsy will be obtained from the vastus lateralis, under local anesthesia (Xylocaine 1%), using the percutaneous needle biopsy technique as previously described [12]. Exercise will commence 10 to 20 minutes after the resting muscle biopsy. The high intensity exercise will be performed as previously described and will consist of a 6-minute warm-up at 50% to 60% of peak heart rate followed by 4 × 4-minute intervals at 90% to 95% peak heart rate (HR_peak_) and a 2-minute warm-down at 50% to 60% of peak heart. The high-intensity intervals will be separated by 2 minutes of active recovery consisting of cycling at 50% to 70% of peak intensity [12]. Peak heart rate will be defined as the highest heart rate measured during the incremental test.

After completing the exercise, participants will recover for 3 hours and blood samples will be taken immediately, 30 minutes, 1, 2, and 3 hours post exercise (Figure 1). Then, a 2-hour euglycaemic-hyperinsulinaemic clamp (insulin clamp) will be performed. During the clamp blood samples (∼1-1.5 ml) will be taken every 5 minutes to monitor blood glucose levels. A 10-ml blood sample will be taken every 30 minutes during the clamp (see Figure 1) for the analysis of OC, ucOC, insulin, and other markers of bone formation and resorption (the hormones CTX and procollagen 1 N-terminal propeptide (P1NP)). Blood will be centrifuged (10 min at 3500 rpm, 4°C) and serum and plasma will be immediately stored in aliquots at −80ºc until assayed. In addition, a total of three muscle biopsies from the vastus lateralis will be undertaken in each experimental session. Muscle will be analyzed for insulin signaling proteins, markers of muscle inflammation and atrophy signaling proteins. In total, seven muscle biopsies will be obtained during the study. Four biopsies will be obtained during the first session (−24, baseline and pre and post insulin clamp) and three during the second session (baseline and pre and post insulin clamp).

A euglycemic-hyperinsulinemic clamp (insulin clamp) will be performed as previously reported [12,27,28]. Prior to the insulin clamp a single slow release tablet of potassium chloride (600 mg) will be given to reduce the risk of potassium depletion during the insulin clamp. Venous blood samples, from a heated arm vein, will be collected prior and during each session. Insulin will be infused at 40 mU.m^-2^per minute for 120 minutes generating an elevated, stable insulin concentration in the last 30 minutes of the clamp. Insulin sensitivity will be assessed by the glucose infusion rate (GIR, mg·kg^-1^·min^-1^) during the last 30 minutes of the insulin-stimulated period and the GIR per unit of insulin (M-Value) [29]. During both euglycemic-hyperinsulinemic clamp sessions, exogenous glucose will be variably infused to achieve the target blood glucose of approximately 5 mmol/L for the duration of the clamp.

### Analysis

Total serum OC will be measured using an automated immunoassay. Serum ucOC will be measured by the same immunoassay after adsorption of carboxylated OC on 5 mg/mL hydroxyl-apatite slurry, following the method described by Gundberg et al [30]. β-isomerized C-terminal telopeptides (a bone resorption marker) and P1NP (a bone formation marker) will be analyzed at Austin pathology.

Muscle insulin signaling proteins, markers of muscle inflammation and atrophy signaling proteins (see below for details) will be measured by PCR and immunoblotting. Glucocorticoid signaling in muscle will be also monitored by quantitative reverse-transcription PCR analysis of glucocorticoid-target genes, *Gilz*and *Fkbp5*. Protein expression of the GILZ and Fkbp5 will also be analyzed by immunoblotting. Muscle mitochondrial respiration will be measured using Oxygraph O2k, to assess the effects of prednisolone and exercise on mitochondrial function.

### Safety Consideration

This is an invasive study that includes seven muscle biopsies (in total), two insulin clamps, blood sampling, high intensity exercise, and administration of a single dose of prednisolone. To reduce the risk associated with the study, only healthy young individuals will participate. In addition, only those who meet the inclusion criteria (as described above) will participate. During the initial graded exercise test participants will be monitored via a 12-lead electrocardiogram (ECG) to identify cardiac abnormalities that may exclude them from the study. A 12-lead ECG will also be used during the insulin clamps. Participants will be asked to report side effects associated with the administration of prednisolone (and placebo). In case of an adverse response/event the participant will be seen by the medical practitioner involved in the study. All the participants will receive a copy of their results (blood tests, body composition, and aerobic fitness).

### Ethical Considerations

This study was approved by Victoria University Human Research Ethics Committee (Application ID: HRE14-099). The trial has been regis­tered in the Australian New Zealand Clinical Trials Registry [ANZCTR, ID ACTRN12610000943044]. The investigator, regardless of the outcome, will publish the results of the study.

### Outcomes Measurements

Primary outcomes are changes in serum ucOC and changes in insulin sensitivity (glucose infusion rate per unit of insulin). Secondary outcomes are measures of skeletal muscle insulin signaling proteins for total and phosphorylated forms of protein kinase B, Akt substrate of 160 kDa, and Insulin receptor substrate 1 and 2, mitochondria function and markers of muscle inflammation including interleukin-6, tumor necrosis factor-alpha, monocyte chemotactic protein 1, interleukin 1 beta, atrogin, signal transducer and activator of transcription 3, nuclear factor kappa-light-chain-enhancer of activated B cells, forkhead box protein O1, and Muscle RING-finger protein-1. In addition, glucocorticoid-target genes, *gilz*and *Fkbp5*will be measured. G protein-coupled receptor class C group 6 member A will also be measured as it is the likely receptor for ucOC in skeletal muscle. In blood, changes in bone remodeling markers include total osteocalcin, P1NP, and beta-isomerized C-terminal telopeptides.

## Discussion

### Principal Findings

In mice, ucOC is a modulator of insulin sensitivity and ucOC treatment reduces the risk for type 2 diabetes [2,3]. In humans, data are supportive but are based on cross-sectional studies. If this study shows that suppression of ucOC with a single dose of prednisolone leads to suppression of insulin sensitivity post exercise, this suggests that ucOC is likely to participate in the modulation of insulin sensitivity in humans.

Insulin resistance is characterized by impaired insulin action in insulin target tissues. However, muscle glucose uptake is normal during and following exercise in patients with type 2 diabetes [31,32]. As such, understanding the mechanism/s behind the insulin sensitizing effect of exercise on muscle and whole-body glucose uptake can open the door for new therapeutic treatments for T2DM. In this study we focus on ucOC as acute exercise increases ucOC and higher ucOC correlate with an improvement in insulin sensitivity and glycemic control after exercise in obese men [7,12,13].

A single dose (10 mg) of prednisone suppresses OC and ucOC, an effect that lasts for at least 12 hours [20]. We hypothesize that prednisolone will attenuate the increase in ucOC in response to exercise and that this will coincide with reduced insulin sensitivity compared with placebo. This design will enable us to examine whether changes in ucOC levels are related to changes in insulin sensitivity in young healthy men. We also aim to determine the fraction of the observed reduction in insulin sensitivity that is modulated by ucOC in humans.

A potential limitation of the current study is that GC are known to effect several other organs/tissues and not only osteoblasts. As such, it is plausible that a reduction in insulin sensitivity following acute GC treatment may not be entirely due to the reduction in ucOC. However, in mice the reduction in ucOC and insulin sensitivity occurred prior to the GC-induced effects in muscle, liver, and adipose following short-term GC treatment suggesting that the changes in insulin sensitivity are related to the reduction in ucOC levels and not to the GC effects on skeletal muscle, fat, or liver [17]. Nevertheless, in the current study we will be able to identify if acute GC treatment has immediate effects on GC-target genes ( *gilz, Fkbp5*, fatty acid binding protein 4, hydroxysteroid (11-Beta) dehydrogenase 1, peroxisome proliferator-activated receptor gamma, and CCAAT/enhancer-binding protein alpha), and any detrimental effect on skeletal muscle, as markers of muscle atrophy and degradation will also be measured.

Another limitation is that the current study will investigate young, healthy men rather than patients with T2DM. As such, it will not be possible to generalize the results to this population. Follow-up studies will be required to confirm that changes in ucOC following interventions are related to changes in insulin sensitivity in patients with T2DM.

### Conclusions

Current evidence connecting ucOC to insulin sensitivity in humans is supportive, but driven by cross-sectional studies. The current dynamic study will add important knowledge concerning the role of ucOC in insulin sensitivity in humans post exercise. The project is relevant to understanding pathway/s involved in exercise-induced insulin sensitivity and the potential for using osteoblast altering drugs as a target to improve insulin sensitivity in diseases like diabetes and obesity.

## Abbreviations

DXA: dual-energy x-ray absorptiometry
ECG: electrocardiogram
Fkbp5: FK506-binding protein 5
GC: glucocorticoid
Gilz: glucocorticoid-induced leucine zipper
GIR: glucose infusion rate
P1NP: procollagen 1 N-terminal propeptide
PCR: polymerase chain reaction
T2DM: type 2 diabetes mellitus
ucOC: undercarboxylated osteocalcin

## References

[1] Yamauchi T, Kamon J, Waki H, Terauchi Y, Kubota N, Hara K, Mori Y, Ide T, Murakami K, Tsuboyama-Kasaoka N, Ezaki O, Akanuma Y, Gavrilova O, Vinson C, Reitman ML, Kagechika H, Shudo K, Yoda M, Nakano Y, Tobe K, Nagai R, Kimura S, Tomita M, Froguel P, Kadowaki T (2001). The fat-derived hormone adiponectin reverses insulin resistance associated with both lipoatrophy and obesity. Nat Med.

[2] Lee NK, Sowa H, Hinoi E, Ferron M, Ahn JD, Confavreux C, Dacquin R, Mee PJ, McKee MD, Jung DY, Zhang Z, Kim JK, Mauvais-Jarvis F, Ducy P, Karsenty G (2007). Endocrine regulation of energy metabolism by the skeleton. Cell.

[3] Ferron M, Hinoi E, Karsenty G, Ducy P (2008). Osteocalcin differentially regulates beta cell and adipocyte gene expression and affects the development of metabolic diseases in wild-type mice. Proc Natl Acad Sci U S A.

[4] Levinger I, Lin X, Zhang X, Brennan-Speranza TC, Volpato B, Hayes A, Jerums G, Seeman E, McConell G (2016). The effects of muscle contraction and recombinant osteocalcin on insulin sensitivity ex vivo. Osteoporos Int.

[5] Liu D, Guo X, Tong H, Tao B, Sun L, Zhao H, Ning G, Liu J (2015). Association between osteocalcin and glucose metabolism: a meta-analysis. Osteoporos Int.

[6] Kunutsor SK, Apekey TA, Laukkanen JA (2015). Association of serum total osteocalcin with type 2 diabetes and intermediate metabolic phenotypes: systematic review and meta-analysis of observational evidence. Eur J Epidemiol.

[7] Levinger I, Zebaze R, Jerums G, Hare DL, Selig S, Seeman E (2011). The effect of acute exercise on undercarboxylated osteocalcin in obese men. Osteoporos Int.

[8] Kanazawa I, Yamaguchi T, Yamauchi M, Yamamoto M, Kurioka S, Yano S, Sugimoto T (2011). Serum undercarboxylated osteocalcin was inversely associated with plasma glucose level and fat mass in type 2 diabetes mellitus. Osteoporos Int.

[9] Brennan-Speranza TC, Conigrave AD (2015). Osteocalcin: an osteoblast-derived polypeptide hormone that modulates whole body energy metabolism. Calcif Tissue Int.

[10] Duncan GE, Perri MG, Theriaque DW, Hutson AD, Eckel RH, Stacpoole PW (2003). Exercise training, without weight loss, increases insulin sensitivity and postheparin plasma lipase activity in previously sedentary adults. Diabetes Care.

[11] Wojtaszewski JF, Hansen BF, Kiens B, Markuns JF, Goodyear LJ, Richter EA, Gade (2000). Insulin signaling and insulin sensitivity after exercise in human skeletal muscle. Diabetes.

[12] Levinger I, Jerums G, Stepto NK, Parker L, Serpiello FR, McConell GK, Anderson M, Hare DL, Byrnes E, Ebeling PR, Seeman E (2014). The effect of acute exercise on undercarboxylated osteocalcin and insulin sensitivity in obese men. J Bone Miner Res.

[13] Kim Y, Nam JS, Yeo D, Kim KR, Suh S, Ahn CW (2015). The effects of aerobic exercise training on serum osteocalcin, adipocytokines and insulin resistance on obese young males. Clin Endocrinol (Oxf).

[14] Cooper MS, Seibel MJ, Zhou H (2016). Glucocorticoids, bone and energy metabolism. Bone.

[15] Henneicke H, Gasparini SJ, Brennan-Speranza TC, Zhou H, Seibel MJ (2014). Glucocorticoids and bone: local effects and systemic implications. Trends Endocrinol Metab.

[16] Moghadam-Kia S, Werth VP (2010). Prevention and treatment of systemic glucocorticoid side effects. Int J Dermatol.

[17] Brennan-Speranza TC, Henneicke H, Gasparini SJ, Blankenstein KI, Heinevetter U, Cogger VC, Svistounov D, Zhang Y, Cooney GJ, Buttgereit F, Dunstan CR, Gundberg C, Zhou H, Seibel MJ (2012). Osteoblasts mediate the adverse effects of glucocorticoids on fuel metabolism. J Clin Invest.

[18] Mittelstadt PR, Ashwell JD (2001). Inhibition of AP-1 by the glucocorticoid-inducible protein GILZ. J Biol Chem.

[19] Davies TH, Ning Y, Sánchez ER (2002). A new first step in activation of steroid receptors: hormone-induced switching of FKBP51 and FKBP52 immunophilins. J Biol Chem.

[20] Nielsen HK, Charles P, Mosekilde L (1988). The effect of single oral doses of prednisone on the circadian rhythm of serum osteocalcin in normal subjects. J Clin Endocrinol Metab.

[21] Short KR, Nygren J, Bigelow ML, Nair KS (2004). Effect of short-term prednisone use on blood flow, muscle protein metabolism, and function. J Clin Endocrinol Metab.

[22] Szulc P, Chapuy MC, Meunier PJ, Delmas PD (1993). Serum undercarboxylated osteocalcin is a marker of the risk of hip fracture in elderly women. J Clin Invest.

[23] Levinger I, Goodman C, Hare DL, Jerums G, Selig S (2007). The effect of resistance training on functional capacity and quality of life in individuals with high and low numbers of metabolic risk factors. Diabetes Care.

[24] Levinger I, Goodman C, Matthews V, Hare DL, Jerums G, Garnham A, Selig S (2008). BDNF, metabolic risk factors, and resistance training in middle-aged individuals. Med Sci Sports Exerc.

[25] Heuck C, Wolthers OD (1998). A placebo-controlled study of three osteocalcin assays for assessment of prednisolone-induced suppression of bone turnover. J Endocrinol.

[26] Kumar N, Crocker T, Smith T, Pow-Sang J, Spiess PE, Connors S, Chornukur G, Dickinson SI, Bai W, Williams CR, Salup R, Fu W (2012). Prostate cancer chemoprevention targeting high risk populations: model for trial design and outcome measures. J Cancer Sci Ther.

[27] Hutchison SK, Stepto NK, Harrison CL, Moran LJ, Strauss BJ, Teede HJ (2011). Effects of exercise on insulin resistance and body composition in overweight and obese women with and without polycystic ovary syndrome. J Clin Endocrinol Metab.

[28] Stepto NK, Cassar S, Joham AE, Hutchison SK, Harrison CL, Goldstein RF, Teede HJ (2013). Women with polycystic ovary syndrome have intrinsic insulin resistance on euglycaemic-hyperinsulaemic clamp. Hum Reprod.

[29] Howlett KF, Mathews A, Garnham A, Sakamoto K (2008). The effect of exercise and insulin on AS160 phosphorylation and 14-3-3 binding capacity in human skeletal muscle. Am J Physiol Endocrinol Metab.

[30] Gundberg CM, Nieman SD, Abrams S, Rosen H (1998). Vitamin K status and bone health: an analysis of methods for determination of undercarboxylated osteocalcin. J Clin Endocrinol Metab.

[31] Kingwell BA, Formosa M, Muhlmann M, Bradley SJ, McConell GK (2002). Nitric oxide synthase inhibition reduces glucose uptake during exercise in individuals with type 2 diabetes more than in control subjects. Diabetes.

[32] Devlin JT, Hirshman M, Horton ED, Horton ES (1987). Enhanced peripheral and splanchnic insulin sensitivity in NIDDM men after single bout of exercise. Diabetes.

